# The Impact of the COVID-19 Pandemic on Ophthalmology Residency and Fellowship Training: A Retrospective Study

**DOI:** 10.7759/cureus.65531

**Published:** 2024-07-27

**Authors:** Abdulmalik Alyahya, Abdulrahman Alyahya, Abdulrahman Alammar, Sami AlShahwan

**Affiliations:** 1 Ophthalmology, King Khaled Eye Specialist Hospital, Riyadh, SAU

**Keywords:** phacoemulsification surgery, ophthalmology, fellow, residency, covid19

## Abstract

Background

The COVID-19 pandemic has negatively affected many aspects of the healthcare system. Many meta-analysis studies showed that surgical training and medical education have faced the most negative effects.

Aim

In this study, we aim to study the impact of the COVID-19 pandemic on residency and fellowship training in terms of clinical load, surgical exposure, medical education, and research opportunities.

Methodology

This retrospective study was conducted at King Khaled Eye Specialist Hospital (KKESH), Riyadh, Saudi Arabia, to assess the effects of COVID-19 on the training program by comparing the years 2018 and 2019 before the COVID-19 pandemic to 2020 and 2021 after the COVID-19 pandemic. The sample was inclusive, including 117 KKESH graduates (residents and fellows in the surgical subspecialties) from 2018 to 2021. All the sample populations were included. The data was collected in a specifically designed form. All participants were ensured to have a full surgical logbook with no missing data in the documentation. Outpatient visits were collected through electronic records in the hospital registry. The teaching activities and grand rounds were collected through the residency and fellowship program.

Results

During 2018-2021, 18,669 surgeries were performed. The total number of surgeries performed was 3,980, 4,898, 4,813, and 4,978 in 2018, 2019, 2020, and 2021, respectively. There was a 23.1% (N = 918) increase in the number of surgeries done by trainees from 2018 to 2019, then a 1.7% (N = 85) reduction from 2019 to 2020, followed by an increase of 3.4% (N = 165) from 2020 to 2021. The mean number of surgeries performed by fellows showed a 25.2% (N = 1,042) increase from 2018 to 2019, a 7.3% (N = 107) reduction from 2019 to 2020, and a 15.1% (N = 550) increase (p = 0.018). In the case of residents, there was a 10.7% (N = 136) reduction from 2018 to 2019, a 2.1% (N = 24) increase from 2019 to 2020, and a 40% (N = 783) reduction in the total number of phacoemulsification surgeries (p < 0.001).In total, there was a 25.1% (N = 8,215) increase in the number of patients seen in outpatient clinics from 2019 to 2020. All activities were on-site during 2018 and 2019. A gradual shift occurred from on-site to virtual over 2020 and 2021 without any effect on the number. From 2019 to 2020, there was an increase in the number of papers submitted by the trainees. There was an increase of 25% (N = 10), 20% (N = 2), and 6.3% (N = 3) in the retrospective research, prospective research, and case reports, respectively.

Conclusions

The surgical exposure has not affected the fellows and residents of the hospital. However, the number of surgeries for the residents has been affected due to the stoppage of overseas surgical courses during the pandemic. The volume of the outpatient clinic has increased after the pandemic, which could be caused by the increased number of referrals to our tertiary hospital, mainly after the pandemic effect on other hospitals in the kingdom and the implementation of the virtual clinic and telephone call. Interestingly, research activity has also increased after the pandemic.

## Introduction

The COVID-19 pandemic has negatively affected many aspects of the healthcare system since the WHO first announced COVID-19 as a pandemic on December 11, 2019 [1]. More serious impacts have surrounded healthcare providers, patients, and the community. The COVID-19 pandemic has not only overwhelmed the healthcare system but also health education [2].

In consideration of medical education, residency and fellowship training have likely been the most affected aspects during the pandemic, as the training requirements demand sufficient exposure to both clinical and surgical experience, which have been reduced due to the pandemic regulations [3,4]. Particularly, ophthalmology residency training and fellowship programs have faced numerous challenges in Saudi Arabia. For instance, some hospitals have suspended their ophthalmology residency training services, both fully and partially. Others have limited their hospital admissions and surgery to emergency cases. In addition, ophthalmological surgical outreach programs and surgical campaigns have been canceled during the pandemic.

In response to the limitations that faced the ophthalmology residency and fellowship programs, the ophthalmology institutes began working on plans and methods to overcome such challenges. The number of virtual grand rounds, conferences, and wet-lap courses has increased [5]. Some institutes have increased the surgical teaching session, and others have arranged an external rotation for their trainees [6].

The aim of this study is to assess the impact of the COVID-19 pandemic on the ophthalmology residency and fellowship training at King Khaled Eye Specialist Hospital (KKESH) and to examine the solutions that have been implemented during the pandemic.

## Materials and methods

This retrospective study was conducted at KKESH, Riyadh, Saudi Arabia. In this study, we assess the effect of COVID-19 on the training program in terms of the ophthalmology clinic loads, number of surgeries, teaching activities, and research projects and publications by comparing the years 2018 and 2019 before the COVID-19 pandemic to 2020 and 2021 after the COVID-19 pandemic. The institutional review board at KKESH approved the current study.

The sample was inclusive, including 117 KKESH graduates (residents and fellows in the surgical subspecialties) from 2018 to 2021. All the sample populations were included. The data was collected in a specifically designed form. All participants were ensured to have a full surgical logbook with no missing data in the documentation. Outpatient visits were collected through electronic records in the hospital registry. The teaching activities and grand rounds were collected through the residency and fellowship program. The research project types and publications were collected through the research department. Then, data cleaning, management, and coding were done using Microsoft Excel 356 (Microsoft Corporation, Redmond, WA, United States).

Statistical analysis

Reduction or increase data were summarized with percentages when quantitative data had a mean, standard deviation, and range. The normality of the data was assessed with the Shapiro-Wilk test. A comparison among more than two groups was performed with a one-way analysis of variance if the data were normally distributed and the Kruskal-Wallis test if the data were not normally distributed. A p-value less than 0.05 was considered statistically significant. Statistical analysis was performed using IBM SPSS Statistics for Windows, Version 22.0 (Released 2013; IBM Corp., Armonk, NY, USA). All figures were created using Microsoft Excel (2019, Microsoft Corporation).

## Results

Surgical exposure

During 2018-2021, 18,669 surgeries were performed. The total number of surgeries performed was 3,980, 4,898, 4,813, and 4,978 in 2018, 2019, 2020, and 2021, respectively. A total of 14,321 surgeries were performed by 84 fellows, and 4,348 surgeries were performed by 33 residents as leading surgeons. There was a 23.1% (N = 918) increase in the number of surgeries done by trainees from 2018 to 2019, then a 1.7% (N = 85) reduction from 2019 to 2020, followed by an increase of 3.4% (N = 165) from 2020 to 2021. Table 1 shows the mean and standard deviation of the number of surgeries performed by residents and fellows in 2018-2021. The mean total number of surgeries done by the trainee showed a 15% increase from 2018 to 2019, a 5.3% reduction from 2019 to 2020, and a 0.6% increase from 2020 to 2021 (p = 0.411). This pattern becomes statistically significant when comparing the resident and fellow surgeries separately. A similar pattern is shown for surgeries performed by fellows: a 25.2% (N = 1,042) increase from 2018 to 2019, a 7.3% (N = 107) reduction from 2019 to 2020, and a 15.1% (N = 550) increase from 2020 to 2021 (p = 0.018). In the case of residents, there was a 10.7% (N = 136) reduction from 2018 to 2019, a 2.1% (N = 24) increase from 2019 to 2020, and a 40% (N = 783) reduction in the number of phacoemulsification surgeries (p < 0.001).

**Table 1:** Number of surgeries performed by residents and fellows from 2018 to 2021 A p-value of 0.05 or less is considered statistically significant.

Year	Total		Fellows		Residents	
	N	Mean ± SD (range)	N	Mean ± SD (range)	N	Mean ± SD (range)
2018	27	147 ± 40 (75-210)	19	143 ± 41 (75-209)	8	159 ± 38 (125-210)
2019	29	169 ± 42 (92-259)	21	179 ± 41 (92-259)	8	142 ± 30 (118-200)
2020	30	160 ± 40 (99-271)	22	166 ± 45 (99-271)	8	145 ± 12 (131-162)
2021	31	161 ± 72 (75-299)	22	191 ± 65 (94-299)	9	87 ± 9 (75-210)
p-value		0.411		0.018		<0.001

**Figure 1:**
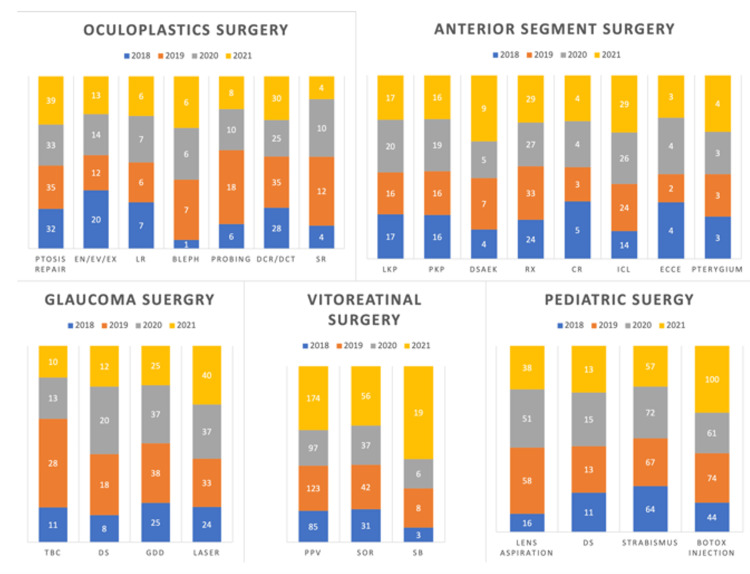
Number of the common surgeries done by fellows in different subspecialties Bleph, blepharoplasty; CR, corneal ring implantation; DCR, dacryocystorhinostomy; DCT, dacryocystectomy; DS, deep sclerectomy; DSAEK, Descemet’s stripping automated endothelial keratoplasty; ECCE, extracapsular cataract extraction; EN/EX/EV, enucleation/exenteration/evisceration; GDD, glaucoma drainage device; ICL, implantable Collamer lens; LKP, lamellar keratoplasty; LR, lid reconstruction; PKP, penetrating keratoplasty; PPV, pars plana vitrectomy; RX, refractive surgery; SB, scleral buckle; SOR, silicon oil removal; SR, socket reconstruction; TBC, trabeculectomy

The number of phacoemulsification surgeries performed per year from 2018 to 2021 showed no statistically significant change before and during COVID-19 for the fellows (p = 0.973). However, there was a 10.7% (N = 136) reduction from 2018 to 2019 and a 40% (N = 783) reduction from 2020 to 2021 (p < 0.001) in the case of residents if we compare the total phacoemulsification surgeries. However, if we compare the phacoemulsification surgeries that were done by the residents in our hospital, there was no statistical difference between the years 2018 and 2021 (Table 2). The number of anterior segment surgeries done by the anterior segment fellows between 2018 and 2021 showed an 18.2% (N = 36) reduction in refractive surgeries and a 36.6% (N = 582) increase in phacoemulsification surgeries from 2019 to 2020. There is no significant change in the number of other procedures. The number of vitreoretinal surgeries between 2018 and 2021 has a 21.1% (N = 156) and 11.9% (N = 30) reduction in pars plana vitrectomy and silicon oil removal from 2019 to 2020, respectively. There was a 53.6% (N = 60) reduction in trabeculectomy surgeries from 2019 to 2020. There was no significant change in the number of other glaucoma procedures done by the fellows. In oculoplastic surgeries, there was a 44.4% (N = 8) and 28.6% (N = 10) reduction in lacrimal probing and dacryocystorhinostomy/dacryocystectomy from 2019 to 2020, respectively. There has been no significant change in the number of other surgeries. In the number of pediatric ophthalmology and strabismus surgeries between 2018 and 2021, there was a 12.1% (N = 21) and 21.3% (N = 39) reduction in lens aspiration and Botox injection from 2019 to 2020, respectively (Figure 1).

**Table 2:** Number of phacoemulsification surgeries performed by residents and fellows from 2018 to 2021 A p-value of 0.05 or less is considered statistically significant.

Year	Fellows			Residents (Leading)			
	N	Leading	Assistant	N	KKESH	India	Total
2018	16	48 ± 37	21 ± 16	8	87 ± 38	72 ± 4	159 ± 38
2019	16	44 ± 33	21 ± 19	8	71 ± 31	71 ± 2	142 ± 30
2020	16	48 ± 43	20 ± 16	8	76 ± 17	69 ± 12	145 ± 12
2021	16	41 ± 35	23 ± 12	9	87 ± 9	0 ± 0	87 ± 9
p-value		0.973	0.749		0.509	<0.001	<0.001

Outpatient clinics in the hospital

Table 3 summarizes the number of patients booked in the outpatient clinics in the hospital between 2018 and 2021. In total, there was a 25.1% (N = 8,215) increase in the number of patients seen in outpatient clinics from 2019 to 2020: 61.6% (N = 633) increase in uveitis, 57.8% (N = 600) increase in oculoplastic, 51.8% (N = 1,633) increase in laser/injection, 50.3% (N = 177) reduction in neuro-ophthalmology, and 36.9% (N = 3,054) increase in anterior segment clinics. The number of no-shows in clinics showed a 17.6% (N = 3,596) reduction in total. However, the number of cancellations showed a 36.1% (N = 9,870) increase in total.

**Table 3:** Number of patients seen, no-show, or canceled in outpatient clinics between 2018 and 2021

Clinic	2018			2019			2020			2021		
	Seen	No-show	Cancellation	Seen	No-show	Cancellation	Seen	No-show	Cancellation	Seen	No-show	Cancellation
Anterior segment	7,058	4,299	6,395	8,285	4,805	7,534	11,339	4,520	10,678	8,411	7,176	7,732
Oculoplastic	1,308	825	1,047	1,038	620	632	1,638	752	1,209	2,665	1,808	2,407
Glaucoma	3,439	2,136	3,050	3,806	2,183	3,339	4,937	1,951	4,476	4,313	3,577	3,747
Retina	9,904	5,544	7,650	7,191	4,025	5,297	8,513	3,694	7,735	6,261	4,713	5,784
Uveitis	808	537	622	1,028	592	755	1,661	689	1,475	1,268	q	1,142
Pediatric and strabismus	3,759	1,604	2,133	3,786	1,818	2,689	4,174	1,186	3,345	3,799	2,155	3,371
Neuro-ophthalmology	306	233	313	352	244	313	175	91	159	382	112	364
Laser/injection	3,458	1,789	3,289	3,150	1,646	3,056	4,783	1,291	4,686	2,539	1,286	2,434
Comprehensive	4,853	4,339	4,383	4,155	3,389	3,757	3,786	1,752	3,479	3,095	3,158	2,923
Total	34,893	21,306	28,882	32,791	19,322	27,372	41,006	15,926	37,242	32,733	25,099	29,904

Teaching activities in the hospital

Table 4 summarizes the number of lectures, clinical teaching rounds (CTRs), and grand rounds in the hospital from 2018 to 2021. All activities were on-site during 2018 and 2019. An immediate shift occurred from on-site to virtual over 2020 and 2021.

**Table 4:** Number of lectures, CTRs, or grand rounds in the hospital CTR, clinical teaching round

Subspeciality	2018		2019		2020		2021	
	On-site	Virtual	On-site	Virtual	On-site	Virtual	On-site	Virtual
Anterior segment	18	0	17	0	5	13	0	16
Oculoplastic	7	0	8	0	1	5	0	10
Glaucoma	3	0	4	0	3	8	0	9
Retina and uveitis	21	0	27	0	8	3	0	18
Pediatric and strabismus	6	0	7	0	4	10	0	9
Neuro-ophthalmology	3	0	3	0	0	3	0	4

Research

From 2019 to 2020, there was an increase in the number of papers submitted by the trainees. There was an increase of 25% (N = 10), 20% (N = 2), and 6.3% (N = 3) in the retrospective research, prospective research, and case reports, respectively (Table 5).

**Table 5:** Number of research carried out by residents and fellows from 2018 to 2021 in the hospital

Research	2018			2019			2020			2021		
	Fellows	Residents	Total	Fellows	Residents	Total	Fellows	Residents	Total	Fellows	Residents	Total
Clinical trial	1	0	1	0	0	0	0	0	0	0	0	0
Prospective	6	0	6	7	1	8	9	1	10	4	3	7
Prospective with some retrospective	2	0	2	1	0	1	0	0	0	0	0	0
Retrospective	23	6	29	30	0	30	32	8	40	15	1	16
Retrospective with prospective	1	0	1	0	0	0	1	0	1	0	0	0
Case report	37	3	40	41	7	48	45	6	51	12	3	15
Review	0	0	0	0	0	0	2	0	2	2	0	2

## Discussion

Our hospital has been affected by the COVID-19 pandemic, as have many other healthcare sectors. The most negative effect was during March 2020 and May 2020, when the hospital announced that outpatient clinic visits and surgery would be limited to emergency cases only. Afterward, the hospital implemented strict infection control in the hospital and resumed routine outpatient clinic visits and elective surgery as usual. However, a number of no-shows during the clinic, surgery cancellations, and absent infected physicians and healthcare staff were unpredictably playing a role in the healthcare and education process.

Multiple studies have shown that surgical exposure for the trainee in surgical residency programs was the most negatively affected part [6-10]. In our study, there was a non-statistically significant reduction in the surgeries done by fellows in 2020, but the number has increased more than in 2019 and 2020 in 2021. In terms of residents, there was a statistically significant reduction in the number of total phacoemulsification procedures by 40% (N = 783) after the COVID-19 pandemic, especially when the surgical course in India stopped during the last months of 2020 and the year 2021. However, the number of phacoemulsification surgeries done by the residents of our hospital has shown an increase in the years 2020 and 2021 compared to 2019. In addition, the mean number of phacoemulsification surgeries done by the resident in our hospital was 87, which exceeded the number of required phacoemulsification surgeries set by the Saudi Commission for Health Specialties by seven surgeries. We believe the reduction in the total number was due to stopping the regular surgical course in India for the senior residents, where every resident usually does more than 70 phacoemulsification surgeries. The residency and fellowship program and the hospital administration have increased the teaching of surgical sessions to compensate for the negative effects of the pandemic. In addition, the residency and fellowship program has regularly assessed the number of surgeries for every trainee individually, and if there is a noticeable decrease in the number of surgeries for a trainee, an extra surgical session was given to the more affected trainee to make sure that every resident will exceed the required number by the Saudi Commission for Health Specialties.

Sim and Nam and Chang et al. have shown a decrease in outpatient clinics during the pandemic by 12% and 26%, respectively [11,12]. On the other hand, we found a significant increase in the number of patients in our hospital after the pandemic by 25.1% (N = 8,215). KKESH is a tertiary hospital and the largest ophthalmology referring center in Saudi Arabia. In addition, the hospital rapidly implemented virtual clinics, telemedicine, and telephone consultations. We believe these factors have contributed to the increase in outpatient clinic volume. Utilizing and shifting to the routine use of virtual technology may improve the healthcare system even in difficult circumstances [13,14]. We recommend studying the role of telemedicine and virtual clinics in patient care and hospital practice.

In our hospital, we have shifted to virtual education immediately. The CTR and grand rounds have, fortunately, not been affected as we continue to do all the meetings virtually. Alahmadi et al. studied resident satisfaction with virtual education in Saudi Arabia, and they reported high satisfaction [1].

During the pandemic, many residents and fellows had more opportunities for research activities and projects, which showed, interestingly, a statistically significant increase in the submitted research by the residents and fellows. Such a finding was the same as what was observed by Aviv-Reuven and Rosenfeld, which showed an increase in different types of publications [15]. We believe that the trainees have taken advantage of their time availability during COVID-19 during the restrictions and lockdown. Furthermore, many conferences and symposiums were virtual, which motivated the researchers to share their work across the globe without travel, cost, or time limitations.

This study has given us an insight into our experience during COVID-19 and its effect on the hospital and the residency training; however, there are some limitations. This is a retrospective study where some cofactors cannot be studied. Also, we could not study the actual effect of telemedicine and virtual clinics as the system categorizes them as routine outpatient clinics.

## Conclusions

The surgical exposure for fellows and residents in the hospital has not been affected. However, the number of surgeries for residents decreased due to the suspension of international surgical courses during the pandemic. The volume of outpatient clinics increased post-pandemic, possibly due to a rise in referrals to our tertiary hospital, the pandemic’s impact on other hospitals in the kingdom, and the implementation of virtual clinics and telephone consultations. Additionally, research activity increased after the pandemic.
